# Predicting side-specific prostate cancer extracapsular extension: a simple decision rule of PSA, biopsy, and MRI parameters

**DOI:** 10.1007/s11255-019-02195-1

**Published:** 2019-06-10

**Authors:** Piotr Zapała, Bartosz Dybowski, Ewa Bres-Niewada, Tomasz Lorenc, Agnieszka Powała, Zbigniew Lewandowski, Marek Gołębiowski, Piotr Radziszewski

**Affiliations:** 1 Department of Urology, Medical University of Warsaw, Lindleya 4, 02-005 Warsaw, Poland; 2 Department of Urology, Roefler Memorial Hospital, Pruszków, Poland; 3 1st Department of Clinical Radiology, Medical University of Warsaw, Warsaw, Poland; 4 Department of Pathology, Medical University of Warsaw, Warsaw, Poland; 5 Department of Epidemiology and Biostatistics, Medical University of Warsaw, Warsaw, Poland

**Keywords:** Extracapsular extension, Locally advanced prostate cancer, Multiparametric magnetic resonance imaging, Nomogram, Predictive model, Prognostic tool

## Abstract

**Objective:**

To develop an easy-to-use side-specific tool for the prediction of prostate cancer extracapsular extension (ECE) using clinical, biopsy, and MRI parameters.

**Materials and methods:**

Retrospective analysis of patients who underwent radical prostatectomy preceded by staging multiparametric MRI of the prostate was performed. Multivariate logistic regression analysis was used to choose independent predictors of ECE. Continuous variables were transformed to categorical ones by choosing threshold values using spline knots or testing thresholds used in previously described models. Internal validation of the rule was carried out as well as validation of other algorithms on our group was performed.

**Results:**

In the analyzed period of time, 88 out of 164 patients who underwent radical prostatectomy met inclusion criteria. ECE was evidenced at radical prostatectomy in 41 patients (46.6%) and in 53 lobes (30.1%). In the multivariate analysis PSA, total percentage of cancerous tissue in cores (%PCa) and maximum tumour diameter (MTD) of Likert 3–5 lesions on MRI were independent predictors of ECE. The following rule for predicting side-specific ECE was proposed: %PCa ≥ 15% OR MTD ≥ 15 mm OR PSA ≥ 20 ng/mL. Internal validation of the algorithm revealed safe lower confidence limits for sensitivity and NPV, proving that model offers accurate risk grouping that can be safely used in decision-making.

**Conclusion:**

The rule developed in this study makes ECE prediction fast, intuitive, and side-specific. However, until validated externally it should be used with caution.

## Introduction

Radical prostatectomy (RP) is associated with excellent cancer-specific survival rates and constitutes one of the two curative standards in patients with organ-confined prostate cancer (PCa). Nerve-sparing technique significantly improves postoperative continence and potency. However, in patients with extracapsular extension (ECE), nerve-sparing RP bears risk of positive surgical margins (PSM). ECE is found in 27–36% men of current RP series [1–6]. However, unilateral ECE is much more common than bilateral, thus one side NVB preservation should be possible in at least 80% patients [1, 5, 6], provided that information on this fact is available before surgery. For these reasons, accurate, side-specific preoperative ECE prediction is necessary. Utilising biopsy and clinical parameters, a number of tools for ECE prediction has been developed and successfully validated [2, 5–7]. Simultaneously, the role of multiparametric magnetic resonance imaging (MP-MRI) has increased in preoperative staging [3]. Radiological assessment of ECE is, however, challenged by poorly distinguished direct signs of ECE. Instead, indirect parameters such as capsule bulging, lesion size, or area of the lesion adhering to the capsule have been shown to have higher accuracy. Despite standardization [8, 9], the role of experience cannot be overestimated [10]. New predictive tools are being developed incorporating MRI-based parameters into models [4, 11, 12]. Apart from accuracy, the ideal tools should be characterized by simplicity and prostate side-specificity. Several previous studies suggested that maximum tumour diameter (MTD) might be used in predicting ECE [13]. Since, according to ESUR guidelines, MTD should be reported during every MP-MRI evaluation [9], its radiologic measurement can be easily implemented in preoperative assessment. Herein we developed an easy-to-use side-specific tool for the prediction of ECE based on basic clinical, biopsy, and MP-MRI parameters.

## Materials and methods

### Patients

Patients who underwent radical prostatectomy preceded by MP-MRI for staging in years 2012–2014 were included into this retrospective analysis. The study was approved by institutional review board. MP-MRI was scheduled in all patients operated at that time in our department. Both urologists and radiologists in our institution were starting getting experience in this imaging technique then. In all cases, PCa was detected with transrectal ultrasound-guided biopsy. None of the patients received androgen deprivation therapy or radiotherapy before surgery. Other exclusion criteria were: distant metastases, transurethral resection of the prostate, technical issues preventing the MRI interpretation, incomplete postprostatectomy pathological report, or lack of report from prostate biopsy. The following clinical variables were identified from medical files: age, clinical tumour stage, serum total prostate-specific antigen (PSA), prostate volume, and PSA density (PSAD). For each lobe of the prostate number of positive cores, percentage of positive cores, percentage of cancer in total biopsy specimen, and Gleason score were identified separately. If no cancer was found in the lobe at biopsy, all biopsy-derived variables for this side were counted as zero. For RP specimen Gleason score, surgical margins status and pathological stage were reported for each side separately. All prostate specimens were analyzed according to the International Society of Urological Pathology guidelines (2005) and the TNM classification.

### Image analysis

Staging MP-MRI was performed on a 1.5-T magnetic resonance system (Ingenia; Philips Healthcare, Best, The Netherlands) characterized by direct digital data sampling using a multichannel phased-array coil and an endorectal coil filled with air. Examinations consisted of T2-weighted MRI, DWI, and dynamic contrast-enhanced imaging (DCE-MRI) was carried out according to the ESUR guidelines always more than 4 weeks after biopsy. Examinations were evaluated by a single radiologist with limited experience in prostate MRI at the beginning of the study, while being aware of clinical patient data. The Prostate Imaging Reporting And Data System (PI-RADS) was used to assess lesions [8]. Every lesion was scored on a 1-to-5-point scale for T2-weighted MRI, DWI, and DCE-MRI separately. Subsequently, an overall score (Likert scale 1–5), was given for every lesion. ECE was suspected in the presence of established MRI criteria such as neurovascular bundle asymmetry, obliteration of the rectoprostatic angle, irregular bulging of the prostatic contour, tumour signal intensity within the periprostatic fat, and overt extracapsular tumour. Lesion diameters were assessed each time on the T2-weighted sequences. MTD was defined as the largest diameter of a lesion. For each side of the prostate, the following variables related to the index lesion were collected: PI-RADS score, MTD, location, and T3 suspicion as assessed by a radiologist.

### Biopsy

Patients were qualified to prostate biopsy based on PSA elevation (> 4 ng/mL) or abnormal DRE. Tru-cut biopsy was performed systemically and guided with transrectal ultrasound. MRI was performed after the biopsy and was not used to guide it. In case of suspicious lesion visible in TRUS, additional targeted cores were collected.

### Histopathology

Prostate biopsy pathological reports were reviewed retrospectively. Only reports containing separate information for both sides of the prostate on Gleason score, number of cores, number of positive cores, and area of biopsy cores invaded by cancer were analyzed.

All samples from radical prostatectomy were evaluated by a single genitourinary pathologist who was aware of clinical information of the patient. Gleason grading was performed according to the modified consensus of the International Society of Urological Pathology in 2005.

### Statistical analysis

Continuous variables are presented as medians with the corresponding interquartile ranges (IQR). Univariate and multivariate logistic regression analyses were performed to identify predictors of ECE. To identify any nonlinear relations with ECE, continuous variables were initially analyzed in nonlinear generalized additive models and presented as splines. In the next step, continuous variables were transformed into categorical variables by setting cut-offs. Thresholds were defined based on spline knots which were identified for most significant gain in predicted ECE risk. If more than one knot was present or knot represented wide range of variable values, cut-off representing lower value was selected to minimalize false negatives. If no nonlinear relation was found for variable, already existing cut-offs that describe D’Amico risk groups were used (PSA: 10–20 ng/mL and > 20 ng/mL; GS = 7 and GS ≥ 8). If no nonlinear relation with ECE was found and variable is not included in D’Amico risk grouping, Youden’s *J* statistic (*J* = sensitivity + specificity − 1) was used for setting threshold value. To construct the final model, variables transformed as above were used. The independent categorized predictors of ECE were identified using multivariate logistic regression analysis. To evaluate calibration of the model Hosmer–Lemeshow test was performed and calibration plots were created. Model was than adjusted for binary decision rules and clinical algorithm was created. Area under receiver-operating characteristic (ROC) curve, sensitivity, specificity, positive (PPV) and negative predictive value (NPV) of the algorithm, and its components were calculated for bootstrapped data to avoid any optimistic bias.

To refer accuracy, parameters of our algorithm to other tools MSKCC nomogram [6] and recently reported its modification including MRI data by Feng et al. [12] were validated in the same patient group.

Analyses were performed using SAS 9.4 software (SAS Institute, Cary, USA). The threshold for significance was set at *P* < 0.05.

## Results

Of the 164 patients who underwent RP at the designated period 88 individuals had MP-MRI performed according to the protocol and met other inclusion criteria. Mean age was 63.5 years (range 49–83) and mean PSA was 10.5 ng/mL (IQR = 6.6). In this group, 41 men (46.6%) had ECE including 10 with seminal vesicle invasion (11.4%). There were three patients (3.4%) with positive lymph nodes. ECE was reported in 53 lobes (30.1%). Positive surgical margins were reported in 30 patients (34.1%). Suspicious lesions (PI-RADS 3-5) in MP-MRI were described in 82 patients (93.2%) and 116 lobes (65.9%). Radiological signs suggesting ECE were found in 30 patients (34.1%) and 37 lobes (21.0%). Sensitivity, specificity, PPV, and NPV of MP-MRI alone in predicting ECE were 41.5%, 88%, 59.5%, and 77.8% respectively. Detailed clinical characteristics are summarized in Tables 1 and 2.

**Table 1:** Clinical and pathological characteristics of the patients

Variables	No. of patients (%)
PSA	
< 4	6 (6.8%)
< 10	62 (70.5%)
10–20	19 (21.6%)
> 20	7 (8%)
Unilateral cancer	41 (46.6%)
Bilateral cancer	47 (53.4%)
Biopsy Gleason score	
≤ 6	41 (46.6%)
3 + 4	26 (29.5%)
4 + 3	10 (11.4%)
8	7 (8%)
≥ 9	4 (4.5%)
PSAD	
< 0.15	25 (28.4%)
0.15–0.2	32 (36.4%)
> 0.2	31 (35.2%)
Clinical stage (DRE and TRUS)	
cT1c	24 (27.3%)
cT2–3	64 (72.7%)
Likert 3–5 lesion in MRI	
Total	82 (93%)
Unilateral	24 (29.3%)
Bilateral	58 (70.7%)
ECE in MRI	
Total	30 (34.1%)
Unilateral	23 (26.1%)
Bilateral	7 (8.0%)
pT	
pT2a-b	18 (20.5%)
pT2c	29 (33.0%)
pT3a	31 (35.2%)
pT3b	7 (8.0%)
pT3bN1	3 (3.4%)
PSM	30 (34.1%)
Final Gleason Score	
≤ 6	16 (18.2%)
3 + 4	33 (37.5%)
4 + 3	18 (20.5%)
8	14 (15.9%)
≥ 9	7 (8.0%)

*PSA* prostate-specific antigen (ng/mL), *PSAD* prostate-specific antigen density (ng/mL^2^), *cT* clinical stage (DRE/TRUS), *ECE* extracapsular extension, *MRI* magnetic resonance imaging, *pT* pathological stage, *PSM* positive surgical margins;

**Table 2:** Preoperative and postoperative characteristics of tumour or cancer

Variable	Number (%)
No of lobes (sides)	176
Preoperative	
Cancer present	128 (72.7%)
%CaP ≥ 15%	61 (35.3%)
Gleason score (biopsy)	
3 + 3	68 (38.9%)
3 + 4	34 (19.4%)
4 + 3	11 (6.3%)
8 or higher	15 (8.5%)
T + (MRI)^a^	116 (65.9%)
MTD (MRI) ≥ 15 mm^a^	51 (29.0%)
ECE (MRI)	37 (21.0%)
Postoperative	
Cancer present	144 (81.8%)
ECE (+)	53 (30.1%)
Gleason score (postoperative)	
3 + 3	25 (14.2%)
3 + 4	57 (32.4%)
4 + 3	27 (15.3%)
8 or higher	35 (19.9%)

*ECE* extracapsular extension, *%CaP* percentage of cancer in biopsy cores, *T+* tumour found on specific examination, *MTD* maximum tumour diameter, *MRI* magnetic resonance imaging

^a^Lesions classified 3, 4, or 5 on Likert scale

In the univariate analysis, PSA, PSAD, abnormal DRE or TRUS, number of positive cores, percent of positive cores, total percentage of cancerous tissue in cores, Gleason score ≥ 7, suspicion of ECE in MP-MRI, and MTD in MP-MRI were significantly associated with ECE (Table 3). In the multivariate analysis, PSA, total percentage of cancerous tissue in cores, and MTD were independent predictors of ECE (Table 3).

**Table 3:** Univariate and multivariate analyses of clinical and pathological factors that predict side-specific prostate cancer extracapsular extension

Variables	Univariate			Multivariate	
	OR^b^ (95% CI)	*p*	AUC	OR^b^ (95% CI)	*p*
Initial model					
PSA	2.9 (1.7–5.0)	0.0002	0.660	3.1 (1.5–6.3)	0.0005
cT+^a^	3.0 (1.5–6.4)	0.0032	0.627		NS
No. of positive cores^a^	1.8 (1.4–2.4)	< 0.0001	0.711		NS
% of positive cores^a^	2.4 (1.6–3.4)	< 0.0001	0.724		NS
% of cancer in cores^a^	2.5 (1.8–3.6)	< 0.0001	0.761	2.2 (1.5–3.5)	< 0.0001
GS^a^	2.2 (1.4–3.3)	0.0004	0.682		NS
ECE on MRI^a^	5.1 (2.4–11)	< 0.0001	0.647		NS
MTD^a^	2.6 (1.8–3.9)	< 0.0001	0.753	1.8 (1.2–2.9)	0.006
Final model^c^					
MTD ≥ 15 mm^a^				7.8 (3.3–18.9)	< 0.0001
% of cancer in cores ≥ 15%^a^				6.9 (2.9–16.3)	< 0.0001
PSA 10–20 ng/mL				1.4 (0.5–3.6)	NS
PSA ≥ 20 ng/mL				16.6 (2.6–105)	0.0028

*OR* odds ratio, *AUC* area under curve, *CI* confidence interval, *PSA* prostate-specific antigen (ng/mL), *PSAD* prostate-specific antigen density (ng/mL^2^), *cT*+ tumour found on digital rectal examination or transrectal ultrasound, *ECE* extracapsular extension, *MRI* magnetic resonance imaging, *GS* Gleason Score, *MTD* maximum tumour diameter in MRI (mm), *NS* not significant

^a^Side-selective variables, ^b^OR normalized to 1 SD, ^c^multivariate model based on independent basic model variables that were categorized

**Fig. 1:**
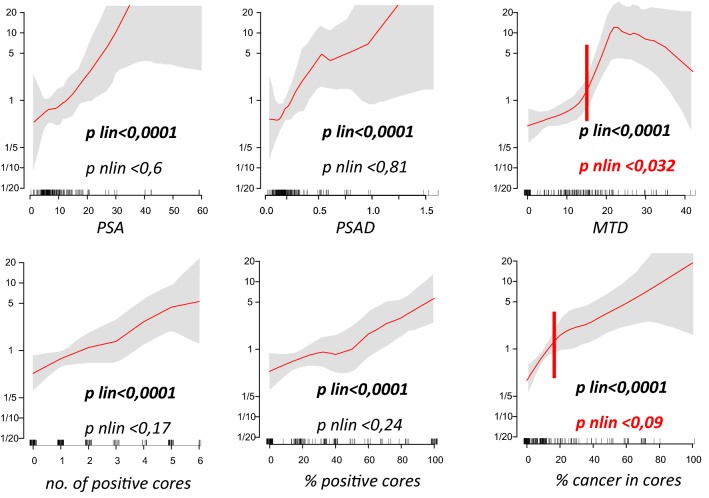
Splines for continuous predictors of extracapsular extension

Spline analysis revealed that MTD and total percentage of cancerous tissue in cores might be associated with ECE in a nonlinear pattern (*P* < 0.03 and *P* < 0.09, respectively). Based on spline knots, cut-offs were identified as 15% for total percentage of cancerous tissue and 15 mm for MTD (Fig. 1). Since no nonlinear relation with ECE was found for PSA, categorization of this variable was based on the cut-off that describes D’Amico risk groups. As a result, we propose the following rule to predict side-specific ECE:

$$ \begin{aligned} &{\text{high risk of right- /left- side ECE if:}} \hfill \\ &\begin{array}{*{20}l} {} \hfill & {\% {\text{PCa in right/left biopsy specimen}} \ge \,15\% } \hfill \\ {\text{OR}} \hfill & \begin{array}{l}{\text{ MTD (MRI) of a lesion Likert 3, 4 or 5}} \ge \,15\; {\text{mm}}\;{\text{in right/left lobe}}\end{array} \hfill \\ {\text{OR}} \hfill & { \, \,{\text{PSA}}\, \ge \,20 \;{\text{ng/mL}} .} \hfill \\ \end{array} \hfill \\ \end{aligned} $$

Since PSA is not side-specific parameter, according to the rule PSA ≥ 20 ng/mL should be interpreted as the presence of ECE in both lobes. In the multivariate analysis this composed parameter appeared to be significantly associated with ECE (Table 3). Eight patients (8.1%) had PSA ≥ 20 ng/mL, 53 patients (60.0%) had %PCa ≥ 15% in left or right lobe, and 43 patients (48.8%) had MTD ≥ 15 mm in left lobe or right lobe.

Hosmer–Lemeshow confirmed goodness of fit (*P* = 0.49). Sensitivity, specificity, PPV, and NPV of the final model (bootstrapped data) as well as for MSKCC nomogram and MSKCC nomogram integrated with MP-MRI (Feng nomogram) are presented in Table 4.

**Table 4:** Sensitivity, specificity, PPV, and NPV of our algorithm in the internal validation using bootstrap method and of Ohori and Feng nomograms applied to our group in predicting cancer extracapsular extension

	Sensitivity (95% CI)	Specificity (95% CI)	PPV (95% CI)	NPV (95% CI)
Model internal validation, bootstrap *n* = 200				
Model^a^	91% (83–92)	74% (65–98)	54% (44–65)	94% (89–99)

	ECE probability cut-off (%)	Sensitivity (%)	Specificity (%)	PPV (%)	NPV (%)
Performance of other models in our group					
Ohori (6)	≥ 10	98	35	37	97
	≥ 20	71	69	47	86
	≥ 30	57	80	52	83
	≥ 40	55	85	59	83
	≥ 50	40	92	65	80
Feng (11)	≥ 10	71	80	58	88
	≥ 20	67	83	61	87
	≥ 30	57	89	67	84
	≥ 40	52	90	69	83
	≥ 50	43	92	67	80

*PPV* positive predictive value, *NPV* negative predictive value, *CI* confidence interval, *ECE* extracapsular extension, *MTD* maximum tumour diameter

^a^ECE present at the specific prostate side if: MTD ≥ 15 mm (side-specific) OR % cancer in cores ≥ 15% (side-specific) OR PSA ≥ 20 ng/mL (not side-specific)

## Discussion

The ECE prediction rule developed in this study is both side-specific and simple. It is based on the parameters of three different tests, thanks to which their values complement each other. The calculated accuracy is similar to the more complex nomograms. The tools developed so far mostly present the ECE risk as a percent value, which means that cut-off value must be also chosen for the risk that we are able to accept. In contrast, our model gives a dichotomous value. Hence, calculating the performance of this test becomes also simple. Most of the nomograms either do not allow assessing ECE risk selectively for the side, or do not use MRI parameters, but only clinical and biopsy-driven.

ECE is the most common of adverse feature found in pathology after RP [14]. Patients with ECE are more likely to have positive surgical margins [15], recur [16], and die from the prostate cancer [17]. Inadequate qualification of patients with ECE to nerve-sparing prostatectomy increases the risk of PSM and results in need for adjuvant or salvage radiotherapy. For this reason, whenever ECE is suspected, wider dissection, possibly with resection of neurovascular bundles, is recommended. To improve preoperative risk assessment, numerous nomograms have been developed. Partin tables which included PSA, Gleason score, and clinical stage was the first tool developed to estimate risk of non-organ-confined PCa. However, the tables were not able to distinguish unilateral from bilateral ECE [7]. This issue was solved in other side-specific tools based on biopsy, PSA, and DRE results [1, 2, 5, 6]. The main advantage of first studies is their sample size and extensive external validation, whereas not implementing radiological data remains the main limitation.

Simultaneously with ECE predictive tools development quality of MP-MRI reporting improved which made it possible to include MRI in active surveillance protocol [18] and surgical planning [3, 9]. Since, in patients without suspicious lesions in MP-MRI, the risk of clinically significant PCa is low, MP-MRI has potential to significantly reduce overtreatment [19]. In patients considered for radical treatment, on the other hand, supplementing standard T2-weighted images with diffusion-weighted imaging (DWI) not only reduces biopsy sampling error [20], but offers additional assessment of tumour aggressiveness [21] and improves staging accuracy [4, 22]. It has been proved that decrease of apparent diffusion coefficient (ADC) is associated with harbouring a poorly differentiated cancer independently of biopsy characteristics [23]. Moreover, ADC seems to correlate negatively with cancerous tissue volume marking large lesions [24].

Advances in functional MRI imaging reduced clinical role of previously developed clinical prognostic models. Gupta et al. pointed out that MP-MRI outperformed Partin tables in tumour staging and might reach 90% NPV for ruling out pT3 [25]. Recent metanalysis indicates, however, that staging based on MRI alone lacks sensitivity in detecting ECE; thus, it can miss many pT3 tumours [26]. Moreover, what can be troublesome in defining utility of staging MP-MRI is its dependence on tumour size and location [27], additional modalities used [4, 22], technical aspects of the scanner [22, 28, 29], use of endorectal coil [30], and, most importantly, experience of the radiologist [31, 32]. Aforementioned factors limit the use of MP-MRI alone in predicting ECE. Instead, MP-MRI possesses great potential to complement the classic clinical parameters used in preoperative staging. It is apparent that MRI has ability to supplement clinical data and improve prediction accuracy on nomograms [11, 12, 33]. Feng et al. reported that both Partin tables and MSKCC nomogram by Ohori et al. can improve their accuracy by adding MP-MRI parameters with AUC increasing from 0.85 to 0.92 and 0.86 to 0.94, respectively [12]. Accuracy of both basic models in the study is worth noting as it is relatively high among the existing studies [1, 6]. In our cohort, MSKCC nomogram expressed AUC of 0.794 increasing to 0.816 after supplementing MP-MRI using model by Feng et al. (http://www.cedars-sinai.edu/PathologicECE) which corresponds with aforementioned literature data. Chen et al. updated Ohori and Partin models with ECE risk score by ESUR observing AUC 0.851 for the final model [11]. Recently Morlacco et al. reported that using staging MP-MRI as adjunct for Partin tables and Cancer of the Prostate Risk Assessment (CAPRA) estimates increases its AUC from 0.6106 to 0.7327 and from 0.6907 to 0.7701, respectively [33].

PCa staging with MP-MRI alone is highly dependent on radiologist’s experience [31, 32]. Expert radiologist can reach sensitivity of over 80% with 96% NPV (non-side-specific), while a colleague from the same institution without experience can be less accurate than DRE [31]. The initial lack of experience in prostate MP-MRI reading may be a reason why in our study MTD was found to be much more informative than radiological signs of ECE in multivariate analysis. Although lesion size is often underestimated [9], MTD measurement seems to be less subjective than radiologist judgment on tumour local spread. Largest tumour diameter has been also previously described as potential pathologic predictor of disease recurrence and local spread [13, 34].

In this retrospective study, we aimed at defining clear group at risk of ECE that could aid decision-making in qualification for side-specific nerve-sparing, focal treatment, or active surveillance. To fulfil this aim and provide easy clinical implementation, we categorized PSA that was an independent predictor of ECE using thresholds according to D’Amico grouping. Using high-risk PSA and cut-offs for MTD and total percentage of cancer in cores defined during non-linearity analysis, we developed a simple rule for predicting side-specific ECE. Internal validation of the algorithm revealed safe lower confidence limits for sensitivity (82.6%) and NPV (89.2%). Since lower confidence limits simulate most pessimistic clinical performance, we assume that model provides accurate risk grouping that can be safely used in decision-making. Validation of MSKCC without and with MP-MRI yielded high sensitivity (71% and 67% respectively) and NPV (86% and 87% respectively) when 20% risk threshold was attached. After supplementing Ohori nomogram with imaging staging, the gain was visible in specificity (69% vs 83%) which again confirms that greatest value of MRI lies at reducing false positives.

Concluding these results, it should be emphasized that our algorithm does not seem to be inferior neither to MSKCC side-specific nomogram [6] nor to its recent update by Feng et al. [12], yet offers easier clinical application. The rule based on three conditions gives straightforward outcome suggesting the presence or lack of ECE, whereas nomograms can be hard to drive decisions when percentage of risk is within “grey zone”. However, any categorical statements are premature as our study is based on a limited number of patients from a single institution. Retrospective design predisposes to selection bias. Biopsy technique (systemic and targeted if required) may also affect results. Both increased and decreased numbers of cores collected may affect the significance of PCa percentage in biopsy. It seems that future developments should resolve this problem [1–3, 5, 6, 12, 33]. Due to analyzed period (2012–2014), ISUP 2005 Gleason score and PIRADS v1 were used in predictive analysis which might cause relatively low predictive value of this variable. High rate of biopsy Gleason score 3 + 3 and much higher final Gleason score suggest that mapping biopsy is insufficient for reliable assessment of tumour aggressiveness. Implementation of prebiopsy MRI and targeted biopsy should improve this issue. Finally, our study lacks external validation, and thus, future investigation is needed to minimize overfitting and validate its clinical performance.

### *Take Home Message*

The rule developed in this study makes ECE prediction fast, intuitive, and side-specific. However, until validated externally, it should be used with caution.

## Data Availability

The data sets used and analyzed during the current study are available from the corresponding author on reasonable request.

## References

[1] Steuber T, Graefen M, Haese A, Erbersdobler A, Chun FK-H, Schlom T (2006). Validation of a nomogram for prediction of side specific extracapsular extension at radical prostatectomy. J Urol.

[2] Satake N, Ohori M, Yu C, Kattan MW, Ohno Y, Miyakawa A (2010). Development and internal validation of a nomogram predicting extracapsular extension in radical prostatectomy specimens. Int J Urol Off J Jpn Urol Assoc.

[3] Boesen L, Chabanova E, Løgager V, Balslev I, Mikines K, Thomsen HS (2015). Prostate cancer staging with extracapsular extension risk scoring using multiparametric MRI: a correlation with histopathology. Eur Radiol.

[4] Giganti F, Coppola A, Ambrosi A, Ravelli S, Esposito A, Freschi M (2016). Apparent diffusion coefficient in the evaluation of side-specific extracapsular extension in prostate cancer: development and external validation of a nomogram of clinical use. Urol Oncol.

[5] Sayyid R, Perlis N, Ahmad A, Evans A, Toi A, Horrigan M (2017). Development and external validation of a biopsy-derived nomogram to predict risk of ipsilateral extraprostatic extension. BJU Int.

[6] Ohori M, Kattan MW, Koh H, Maru N, Slawin KM, Shariat S (2004). Predicting the presence and side of extracapsular extension: a nomogram for staging prostate cancer. J Urol.

[7] Partin AW, Mangold LA, Lamm DM, Walsh PC, Epstein JI, Pearson JD (2001). Contemporary update of prostate cancer staging nomograms (Partin Tables) for the new millennium. Urology.

[8] Barentsz JO, Richenberg J, Clements R, Choyke P, Verma S, Villeirs G (2012). ESUR prostate MR guidelines 2012. Eur Radiol.

[9] Barentsz JO, Weinreb JC, Verma S, Thoeny HC, Tempany CM, Shtern F (2016). Synopsis of the PI-RADS v2 guidelines for multiparametric prostate magnetic resonance imaging and recommendations for use. Eur Urol.

[10] Wibmer A, Vargas HA, Donahue TF, Zheng J, Moskowitz C, Eastham J (2015). Diagnosis of extracapsular extension of prostate cancer on prostate MRI: impact of second-opinion readings by subspecialized genitourinary oncologic radiologists. AJR Am J Roentgenol.

[11] Chen Y, Yu W, Fan Y, Zhou L, Yang Y, Wang H (2017). Development and comparison of a Chinese nomogram adding multi-parametric MRI information for predicting extracapsular extension of prostate cancer. Oncotarget.

[12] Feng TS, Sharif-Afshar AR, Wu J, Li Q, Luthringer D, Saouaf R (2015). Multiparametric MRI improves accuracy of clinical nomograms for predicting extracapsular extension of prostate cancer. Urology.

[13] Mizuno R, Nakashima J, Mukai M, Ookita H, Nakagawa K, Oya M (2006). Maximum tumor diameter is a simple and valuable index associated with the local extent of disease in clinically localized prostate cancer. Int J Urol.

[14] Mullins JK, Feng Z, Trock BJ, Epstein JI, Walsh PC, Loeb S (2012). The impact of anatomical radical retropubic prostatectomy on cancer control: the 30-year anniversary. J Urol.

[15] Tollefson MK, Karnes RJ, Rangel LJ, Bergstralh EJ, Boorjian SA (2013). The impact of clinical stage on prostate cancer survival following radical prostatectomy. J Urol.

[16] Eminaga O, Hinkelammert R, Titze U, Abbas M, Eltze E, Bettendorf O (2014). The presence of positive surgical margins in patients with organ-confined prostate cancer results in biochemical recurrence at a similar rate to that in patients with extracapsular extension and PSA ≤ 10 ng/ml. Urol Oncol.

[17] Mikel Hubanks J, Boorjian SA, Frank I, Gettman MT, Houston Thompson R, Rangel LJ (2014). The presence of extracapsular extension is associated with an increased risk of death from prostate cancer after radical prostatectomy for patients with seminal vesicle invasion and negative lymph nodes. Urol Oncol Semin Orig Investig.

[18] Sahibzada I, Batura D, Hellawell G (2016). Validating multiparametric MRI for diagnosis and monitoring of prostate cancer in patients for active surveillance. Int Urol Nephrol.

[19] An JY, Sidana A, Holzman SA, Baiocco JA, Mehralivand S, Choyke PL (2018). Ruling out clinically significant prostate cancer with negative multi-parametric MRI. Int Urol Nephrol.

[20] Turo R, Horsu S, Calinciuc A, Smolski M, Thygesen H, Doyle G, Gulur DM, Das S, Pettersson B, Awsare N (2018). Is magnetic resonance imaging helpful in detecting significant prostate cancer in patients with haematospermia, normal prostate specific antigen level and digital rectal examination. A single institution, observational, and retrospective study in a United Kingdom hospital. Cent Eur J Urol.

[21] Park SY, Oh YT, Jung DC, Cho NH, Choi YD, Rha KH (2017). Diffusion-weighted imaging predicts upgrading of Gleason score in biopsy-proven low grade prostate cancers. BJU Int.

[22] Woo S, Cho JY, Kim SY, Kim SH (2015). Extracapsular extension in prostate cancer: added value of diffusion-weighted MRI in patients with equivocal findings on T2-weighted imaging. AJR Am J Roentgenol.

[23] De Cobelli F, Ravelli S, Esposito A, Giganti F, Gallina A, Montorsi F (2015). Apparent diffusion coefficient value and ratio as noninvasive potential biomarkers to predict prostate cancer grading: comparison with prostate biopsy and radical prostatectomy specimen. AJR Am J Roentgenol.

[24] Bae H, Yoshida S, Matsuoka Y, Nakajima H, Ito E, Tanaka H (2014). Apparent diffusion coefficient value as a biomarker reflecting morphological and biological features of prostate cancer. Int Urol Nephrol.

[25] Gupta RT, Faridi KF, Singh AA, Passoni NM, Garcia-Reyes K, Madden JF (2014). Comparing 3-T multiparametric MRI and the Partin tables to predict organ-confined prostate cancer after radical prostatectomy. Urol Oncol.

[26] de Rooij M, Hamoen EHJ, Witjes JA, Barentsz JO, Rovers MM (2016). Accuracy of magnetic resonance imaging for local staging of prostate cancer: a diagnostic meta-analysis. Eur Urol.

[27] Feng TS, Sharif-Afshar AR, Smith SC, Miller J, Nguyen C, Li Q (2015). Multiparametric magnetic resonance imaging localizes established extracapsular extension of prostate cancer. Urol Oncol.

[28] Heijmink SWTPJ, Fütterer JJ, Hambrock T, Takahashi S, Scheenen TWJ, Huisman HJ (2007). Prostate cancer: body-array versus endorectal coil MR imaging at 3 T—comparison of image quality, localization, and staging performance. Radiology.

[29] Lawrence EM, Gallagher FA, Barrett T, Warren AY, Priest AN, Goldman DA (2014). Preoperative 3-T diffusion-weighted MRI for the qualitative and quantitative assessment of extracapsular extension in patients with intermediate- or high-risk prostate cancer. AJR Am J Roentgenol.

[30] Fütterer JJ, Engelbrecht MR, Jager GJ, Hartman RP, King BF, Hulsbergen-Van de Kaa CA (2007). Prostate cancer: comparison of local staging accuracy of pelvic phased-array coil alone versus integrated endorectal-pelvic phased-array coils. Local staging accuracy of prostate cancer using endorectal coil MR imaging. Eur Radiol.

[31] Renard-Penna R, Rouprêt M, Comperat E, Ayed A, Coudert M, Mozer P (2013). Accuracy of high resolution (1.5 tesla) pelvic phased array magnetic resonance imaging (MRI) in staging prostate cancer in candidates for radical prostatectomy: results from a prospective study. Urol Oncol Semin Orig Investig.

[32] Tay KJ, Gupta RT, Brown AF, Silverman RK, Polascik TJ (2016). Defining the incremental utility of prostate multiparametric magnetic resonance imaging at standard and specialized read in predicting extracapsular extension of prostate cancer. Eur Urol.

[33] Morlacco A, Sharma V, Viers BR, Rangel LJ, Carlson RE, Froemming AT (2017). The incremental role of magnetic resonance imaging for prostate cancer staging before radical prostatectomy. Eur Urol.

[34] Rose BS, Chen M-H, Zhang D, Hirsch MS, Richie JP, Chang SL (2014). Maximum tumor diameter and the risk of prostate-specific antigen recurrence after radical prostatectomy. Clin Genitourin Cancer.

